# Psychological reactions to the coronavirus pandemic: a comparative study of Holocaust survivors and other older adults in Israel

**DOI:** 10.1186/s12888-022-04052-5

**Published:** 2022-06-28

**Authors:** Sara Carmel, Yaacov G. Bachner, Ella Cohn-Schwartz

**Affiliations:** 1 Center for Multidisciplinary Research in Aging, Ben-Gurion University of the Negev, P.O. Box 653, 8410501 Beer-Sheva, Israel; 2 Department of Public Health, Ben-Gurion University of the Negev, Be’er Sheva, Israel

**Keywords:** COVID-19, Anxiety, Older adults, Mental health, Coping resources, Holocaust survivors

## Abstract

**Background:**

The current study examines psychological reactions to the COVID-19 pandemic among older adults living in Israel. Based on the ‘life events, stress, coping and health theory,’ we hypothesized that due to their traumatic early life history and dearth of emotional and physical coping resources, Holocaust survivors would be more vulnerable than other older adults to the negative effects of this difficult and prolonged life event on their mental health.

**Methods:**

Based on structured questionnaires with closed questions, we interviewed 306 Holocaust survivors and non-survivors aged 75 + during the COVID-19 pandemic.

**Results:**

Univariate data analysis showed that Holocaust survivors had fewer coping resources in terms of health status and educational level than non-survivors. As expected, Holocaust survivors also reported a greater extent of COVID-19-related anxiety, and more depression, which worsened during the pandemic. However, both groups did not differ in their will to live, which is an indicator of general well-being and commitment to continue living. In multivariate analyses conducted to explain COVID-19 anxiety in the entire sample and separately on each of the two groups, the best explanatory variables were other psychological variables especially increased depression.

**Conclusions:**

It seems that Holocaust survivors are more emotionally vulnerable to the pandemic’s negative effects than other older adults, in support of the ‘life events, stress, coping and health theory,’ but despite this, they show resilience in their will to continue living. Policy makers and practitioners are recommended to identify Holocaust survivors and other vulnerable older people and investigate their specific needs. Interventions should include practices for maintaining and boosting resilience and well-being by increasing appropriate emotional and cognitive internal and external coping resources, especially during prolonged periods of hardship.

## Background

Since the beginning of the year 2020, the Coronavirus disease (COVID-19) has been a worldwide health and life-threatening pandemic. Not surprisingly, this new, invisible, unscented, untouchable, yet very contagious virus has led to increased uncertainty, worries, and anxiety. Due to lack of sufficient scientific knowledge along with political and social pressures, the preventive measures suggested or enforced by the authorities have been radical and erratic, further increasing uncertainty and anxiety. Among other measures, the Israeli government enforced several general shutdowns lasting up to numerous weeks. During this global, life-threatening and prolonged event, infected older adults have been at the highest risk in the population for developing severe symptoms, often resulting in death.

### The COVID-19 pandemic among older people

The most difficult preventive behavior for social human beings is keeping physical distance from one another, especially from close family members. This is particularly difficult for older persons, who perceive themselves as the population at highest risk for being infected by the COVID-19 virus and are therefore more likely to adopt the suggested preventive behaviors of physically distancing from others [1]. This is especially difficult as many of these older people are community dwellers who live alone. In Israel, 22.6% of people aged 65 and older live alone, as do 31.1% of those aged 75 + [2]. The increased danger of being infected by the disease combined with the necessity to stay isolated at home (quite often alone) with no physical contact even with close family or neighbors, has become particularly difficult for older people [3, 4]. In addition, any necessary contact or interaction with others can cause fear of being infected. This condition of perceived danger and uncertainty on the one hand, and the behavioral limitations which deprive people from social contacts, cultural activities, and often cause delays in treatment of other medical conditions on the other hand, is likely to cause emotional problems such as fear, anxiety, loneliness, insomnia, lack of appetite and other psychosomatic symptoms (e.g., [4, 5]). Such emotional problems may increase decline in mental health and severity of existing chronic diseases. According to the classic 'life events and health theory,' the longer people live under such stressful conditions, the more the negative emotional and physical outcomes may intensify [6].

### Negative life events and overall well-being

Since the 1960’s scientists have investigated the relationship between emotional problems and health [6, 7]. They suggested that exposure to clusters of life events (positive or negative) during a relatively short period of time results in health problems. Other scientists have shown that facing negative life events is especially stressful and detrimental to health (e.g., [8–10]). The assumption behind this relationship is that the exposure to life events demands adjustment to life changes, a stressful psychophysiological process which requires adaptive reactions on the physiological, psychological, and behavioral levels [11, 12], including recruitment and management of mental, physical and environmental resources. If the stressful events and coping process persist, this may lead to a deficit in coping resources followed by increased vulnerability to diseases and even to extreme exhaustion of the body expressed in the form of illnesses and mortality [6, 13]. “*The particular type of health change… depends on the individual’s particular constitutional and acquired weaknesses as well as his exposure to various etiologic agents of disease"* (6, p. 365). According to this theory and further research, the danger to one’s mental and/or physical health depends on the degree of perceived threat to life and/or functioning from the specific life events, the length of exposure to them, the level of predictability or ambiguity related to events, the accumulation of numerous life events during a relatively short period of time, and the extent of specific vulnerabilities or resilience of every individual (e.g., [11, 14]). Personal resources may include various types: physical, psychological, psychosocial abilities, education level and economic abilities, as well as social support at the micro and macro societal levels, all of which may assist in coping with threatening and long-lasting life events. Accordingly, adjustment to stressful life events of people with a traumatic past and fewer resources, such as Holocaust Survivors (HS), may be less effective than that of other older adults, putting them at higher risk for being negatively affected on the emotional level.

On the societal level, various groups of people experience distressing life events in different ways due to the perceived degree of threat imposed by the event, available physical, mental and environmental resources, and psycho-behavioral abilities for effectively managing the necessary coping process following exposure to any negative life event. Hence, weaker social groups in terms of socioeconomic and psychophysical resources such as HS, people in lower social classes, new immigrants, refugees, and people who are physically handicapped, or socially discriminated against, etc., have by definition fewer coping resources compared to other population groups and are therefore more prone to be negatively affected by any societal crisis (e.g., [15, 16]). The current COVID-19 pandemic can be considered a continued threatening life event with potential negative mental and physical health outcomes, especially for older people exposed to traumatic events in their youth, such as HS.

### Holocaust survivors and overall well-being

Currently, HS constitute more than one third of Israelis 75 + years of age [17] The vast majority are very old (80 + years). This number is rapidly declining due to mortality. HS were exposed to especially difficult and horrific events during World War II and came to Israel as indigent refugees. It is interesting to note that comparative studies on HS and their counterparts support two opposing hypotheses: either increased vulnerability and/or increased resiliency (e.g., [17, 18]). In support of the vulnerability hypothesis, some studies have reported that as a group HS, and even their offspring, suffer more than their counterparts from increased morbidity, and from specific chronic diseases such as cardiovascular diseases and/or their risk factors, cancer, osteoporosis, diabetes (e.g., [19–24]), and mental problems including posttraumatic stress (e.g., [25–27]). However, other studies and often even the same studies report that the survivors are a relatively resilient group in terms of certain physical or mental resources (e.g., [18, 26–30]).

In sum, current epidemiological knowledge regarding the COVID-19 pandemic, the life events and health theory, and previous empirical findings supporting it, lead us to assume that the present and ongoing COVID-19 pandemic threatens older population groups more than it does other groups, and has already led to increased mortality among older people. In the present study, we compared two groups of older adults—HS versus other older adults, and hypothesized that the HS, although very young during World War II, and as such not always aware of the horrors taking place around them, would still be a vulnerable social group in terms of psychosocial and physical resources, due to the traumatic circumstances of their youth. We expected that HS would suffer more than other older adults living in Israel from COVID-19-related decline in mental health in terms of anxiety, depression, and general well-being as evaluated by their commitment to continue living – their will to live (WTL). The additional purpose of this study was to investigate the unique contribution of physical, intellectual, and emotional personal resources such as health status, education, depression and WTL as explanative factors of COVID-19-related anxiety in each of the two study groups.

## Methods

### Participants and procedure

We collected data in Israel from 306 older Israelis aged 75 + during the COVID-19 pandemic—between June and November 2020. Of those, 116 were HS and 190 were adults who did not directly experience the Holocaust. HS survivors were defined as residents of European or North-African countries which were occupied or controlled by the Nazis or pro-Nazi regimes during World War II (1939–1945). Non-HS were defined as people who were born in other countries or in Israel. Inclusion criteria were: being 75 years and older and able to understand the study questionnaire in Hebrew.

Subjects were approached by a professional survey agency which contacted participants randomly using a registry-based list and names of HS from social clubs for HS (maintained by a nonprofit organization for the benefit of HS). Although the sample might not be representative, this method allowed us to reach participants from all over the country, decreasing selection bias. In accordance with the COVID-19 regulations, all participants were interviewed via telephone by experienced interviewers. At the beginning of the conversation, participants were advised of the objectives of the study, that participation was completely voluntary, that participants could leave the study at any time, and that the gathered data would be used anonymously for research purposes only. Those who agreed to take part in the study also declared informed consent. Interviews lasted for about 20 min on average. The study was approved by the ethics committee of the University.

### Measures

The questionnaire included socio-demographic and health status questions, as well as questions regarding concerns about being infected by the COVID-19 virus. Socio-demographic details included age (years), gender (female = 1, male = 2) and education. The latter was recoded into two categories of education – elementary and secondary (0–12 years), and higher education (13 + years). Self-rated health was measured by one item “Generally, how would you rate your health status?” with a six-point scale ranging from 1- “excellent” to 6- “very bad”. Responses were reverse coded so that a higher response indicated better health. Corona-related anxiety was assessed by responses to two questions “To what extent are you anxious about contracting the Corona virus?” and “To what extent are you anxious that people close to you will contract the Corona virus?” Possible responses for both items ranged on a five-point scale from 1- “to a very small extent” to 5- “to a very great extent”. The overall score was calculated as the average score for both items, so that a high score indicates a high degree of anxiety. The Pearson correlation coefficient between the two items was high (*r* = 0.69). The Skewness = -0.289 and Kurtosis = -0.799 values indicated a normal distribution. The WTL Scale includes 5 items with 6 possible responses on Likert scales ranging from 0 (no WTL) to 5 (very strong WTL). The index is based on the average score of the responses to the 5 items [31]. Changes in the WTL during the COVID-19 pandemic was measured using a single item with 6 options for response: “During the Corona epidemic would you say that your WTL was: 0- I had no WTL, 1- strongly weakened, 2- weakened, 3- not changed, 4- strengthened, 5- strongly strengthened." This variable was recoded into three categories: WTL has strengthened, WTL has not changed, and WTL has weakened (the latter included the single participant who responded that he had no will to live – 0.3% of the sample). Feelings of depression and anxiety were measured by the item “Have you ever suffered from feelings of depression or anxiety in the past?” with a five-point response scale ranging from 1- “not at all” to 5- “very often”. This item was followed by the question “Would you say that since the Corona crisis these feelings have” 1- “strongly strengthened” 2- “strengthened” 3- “not changed” 4- “weakened” 5- strongly weakened”? We combined these two questions into a single measure with three categories – not having experienced feelings of depression or anxiety (as indicated by the first question), feelings of depression and anxiety haven't changed or have weakened, feelings of depression or anxiety have strengthened during the pandemic (as indicated by the second question).

### Statistical analyses

Descriptive statistics were used to describe the data (means, SD, range). The differences between Holocaust survivors and other older Israelis were examined using Chi square and t-tests, according to two different scale structures (nominal or continuous). The associations between COVID-19 anxiety and the study variables were examined using t-tests or Pearson correlations for dichotomous or continuous variables, respectively. To determine the unique contribution of each of the independent variables to the explanation of participants’ anxiety of being infected with the COVID-19 virus, linear multiple regression analyses were employed. Statistical analyses were performed using SPSS software, version 26. A significant *p*-value was defined as *p* < 0.05.

## Results

Characteristics of the study participants are presented in Table 1. The average respondent age was 81.6 (SD = 5.8), over half of the respondents were women and about 62% of the participants had an above high-school level of education. Thirty-eight percent of the interviewees were Holocaust survivors (HS). Participants reported a medium average level of COVID-19-related anxiety (about 3 out of 5), but a more in-depth examination of their responses showed that 71.7% reported that they were anxious of contracting the Corona virus to a very great/great/somewhat extent (45% of them reported being anxious to a very great or great extent). COVID-19 related anxiety for people close to respondents was even higher (78.6% and 54.3, respectively). Participants perceived their health as satisfactory (4 out of 6). The WTL score was 3.4 (range: 0–5). Regarding WTL, 64.7% of the participants reported no change in their WTL during the COVID-19 pandemic, 22% reported a weakening in their WTL, while 14% reported a strengthening in their WTL. In relation to depression, 42% of the participants reported not having experienced depression or anxiety in the past, 38% experienced depression, but it either did not change or it decreased during the pandemic, and about 20% reported experiencing depression or anxiety during their lifetime, with worsening during the pandemic.

**Table 1:** Descriptive statistics of the sample and bivariate associations between the two study groups

**Variables**	**Total sample**			
		**Non Holocaust Survivors group (*N* = 190, 62%)**	**Holocaust Survivors (*N* = 116, 38%)**	***Bivariate tests***
	**N (%) \ M (SD)**	**N (%) \ M (SD)**	**N (%) \ M (SD)**	
Age	81.6 (5.8)	79.9 (5.4)	84.2 (5.6)	t = -6.414 *p* < 0.001
Gender:				${\chi }^{2}=0.09$
Female	167 (54.9%)	102 (54.3%)	65 (56.0%)	*p* = 0.762
Male	137 (45.1%)	86 (45.7%)	51 (44.0%)	
High education (13 + years)	187 (61.7%)	129 (68.6%)	58 (50.4%)	${\chi }^{2}=9.98$ *p* = 0.001
COVID-19 anxiety	3.4 (1.2)	3.3 (1.3)	3.7 (1.1)	t = -2.92 *p* = 0.004
Experiencing COVID-19 anxiety to a very great\great extent	129 (42.7%)	64 (33.9%)	65 (57.5%)	${\chi }^{2}=16.18$ *p* < .001
Self-perceived health	3.9 (1.1)	4.1 (3.4)	3.4 (4.1)	t = 5.372 *p* < 0.001
Depression:	129 (42.4%)	87 (46.3%)	42 (36.2%)	${\chi }^{2}=6.38$ *p* = 0.041
None\no change	115 (37.8%)	72 (38.3%)	43 (37.1%)	
Increased	60 (19.7%)	29 (15.4%)	31 (26.7%)	
WTL scale	3.4 (0.6)	3.5 (0.7)	3.4 (0.6)	t = 0.497 *p* = 0.155
WTL change:				
WTL weakened	66 (22.0%)	38 (20.2%)	28 (25.0%)	${\chi }^{2}=0.95$
WTL no change	194 (64.7%)	124 (66.0%)	70 (62.5%)	*p* = 0.621
WTL strengthened	40 (13.3%)	26 (13.8%)	14 (12.5%)	

*WTL * Will to live

A comparison between HS and other older Israelis showed that HS were, on average, older and less educated compared to the other group. They also reported higher COVID-19 anxiety, worse self-perceived health, and were more likely to report becoming more depressed during the COVID-19 pandemic. However, they did not differ in gender nor in their WTL average score or their WTL changes during the pandemic (Table 1).

 Results of bivariate associations with COVID-19 anxiety are presented in Table 2. The findings indicate that participants who reported higher levels of COVID-19 anxiety were older, less educated, and more likely to be women. They also rated their health as worse and were more likely to report an increase in their depression and a weakening in their WTL.

**Table 2:** Bivariate associations between the study variables and COVID-19 Anxiety (continuous*)*

Variables	Bivariate tests
Age	*r* = 0.140, *p* < 0.016
Gender	*t* = 2.04, *p* = 0.042
Education	*t* = 2.385, *p* = 0.018
Self-perceived health	*r* = -0.206, *p* < 0.001
Depression change: No depression or weakened No change Increased	*F* = 19.11, *p* < 0.001
WTL scale	*r* = -0.052, *p* = 0.369
WTL change: WTL weakened WTL no change WTL strengthened	*F* = 4.72, *p* = 0.010

*WTL* Will to live

We next performed hierarchical regression models of COVID-19 anxiety using data from the entire sample. The first model included the survivor status and socio-demographic variables of age, gender, education and self-rated health. The results indicate that being HS does not correlate significantly with COVID-19 related anxiety, while perceiving one's health as better was related to lower anxiety. This model was significant (F = 4.290, *p* = 0.001) and explained 7% of the variance in COVID-19 anxiety. We added WTL, change in WTL, and change in depression to the second model. This model was also statistically significant (F = 5.495, *p* < 0.001). The results show that adults who reported a decline in their WTL or an increase in depression were also more anxious about being infected with COVID-19. This model added 9.5% to the explained variance by the first model and explained about 17% of the variance in COVID-19 anxiety of the total sample.

Following this, we used a separate analysis of the data for HS and for the other group, using hierarchical regression models of COVID-19 anxiety (Table 3). In the first step we included the socio-demographic variables of age, gender, education and self-rated health, which, contrary to the results of the associations between the variables in the entire sample, in the group of HS (Table 4) had no statistically significant associations with COVID-19-related anxiety (F = 0.875, *p* = 0.482). In the next step, we added WTL, change in WTL and change in depression. The model in the second step was marginally significant (F = 1.911, *p* = 0.059). The results show that HS who reported an increase in depression were also more anxious about being infected with COVID-19. This model explained about 15% of the variance in COVID-19 anxiety and added 12% to the explained variance of the first model. The results of the first step among the non-HS (who were a larger group) showed that gender was associated with COVID-19-anxiety, so that men reported lower anxiety compared to women. The model in the first step was statistically significant (F = 3.766, *p* = 0.006), accounting for 8% of the variance, but only gender was found to have a significant contribution to the explanation of the variance on COVID-19 anxiety. The results in the second step indicate that the addition of WTL, change in WTL and change in depression added about 9% to the explained variance of COVID-19 anxiety, indicating that adults whose WTL weakened and who reported an increase in depression during the pandemic, also reported more COVID-19-anxiety, while the contribution of gender became only marginally significant. This model was statistically significant (F = 3.853, *p* < 0.001).

**Table 3:** Hierarchical regression models on COVID-19 Anxiety (all participants)

Variable	B	SE	Beta	*p*	B	SE	Beta	*p*
Holocaust survivor	.257	.152	.107	.091	.179	.146	.075	.223
Age	.005	.013	.024	.705	.008	.012	.041	.512
Gender^a^	-.204	.137	-.088	.137	-.103	.133	-.044	.442
High education^b^	-.175	.148	-.073	.240	-.115	.144	-.048	.426
Self-perceived health	-.152	.066	-.141	.023	-.109	.067	-.101	.104
WTL scale					.078	.107	.044	.466
WTL weakened^c^					.356	.166	.128	.033
WTL strengthend^c^					-.020	.197	-.006	.918
No change in depression^d^					-.007	.147	-.003	.965
More depression^d^					.808	.181	.280	.000
R^2^	0.071				0.166			

*WTL* Will to live;

^a^Reference: Female

^b^Reference: Less than high education

^c^Reference: No change in WTL

^d^Reference: Never was depressed

**Table 4:** Hierarchical regression models on COVID-19 anxiety

	*Holocaust survivors*								*Non-Holocaust survivors*								
Variable	B	SE	Beta	*p*	B	SE	Beta	*p*		B	SE	Beta	*p*	B	SE	Beta	*p*
Age	-.017	.023	-.074	.472	-.009	.024	-.039	.711		.018	.015	.092	.231	.017	.015	.085	.255
Gender^a^	.089	.255	.035	.729	.250	.250	.099	.320		-.367	.158	-.171	.021	-.299	.156	-.139	.057
High education^b^	-.224	.262	-.089	.395	-.200	.257	-.080	.438		-.173	.177	-.074	.332	-.077	.175	-.033	.663
Self-perceived health	-.180	.119	-.152	.132	-.139	.120	-.117	.250		-.130	.079	-.122	.102	-.093	.081	-.087	.254
WTL scale					.163	.227	.074	.475						.040	.120	.026	.740
WTL weakened^c^					.255	.301	.089	.399						.404	.200	.152	.045
WTL strengthend^c^					-.243	.365	-.066	.508						.144	.233	.047	.538
No change in depression^d^					.143	.286	.055	.617						-.074	.170	-.033	.664
More depression^d^					1.000	.309	.356	.002						.685	.227	.234	.003
R^2^	0.033				0.151					0.079				0.169			

*WTL* Will to live;

^a^Reference: Female

^b^Reference: Less than high education

^c^Reference: No change in WTL

^d^Reference: Never was depressed

## Discussion

During the COVID-19 pandemic we collected data from 306 HS and other older Israelis, aged 75 + . As based on the life events, stress, coping and health theory as well as on previous reports, we hypothesized that HS are still a relatively more vulnerable (mentally and physically) social group in Israel, having experienced extreme trauma during childhood and/or adolescence, having fewer personal resources in relation to areas such as health, functioning (mental and physical), and education, and therefore HS would suffer from more negative mental outcomes due to exposure to the COVID-19 pandemic than would the other group of older adults. The results of univariate analysis support this hypothesis, as the HS group scored significantly lower on the personal resources of education level and self-perceived health, and suffered more than their counterparts during the pandemic from decline in mental health as assessed by a higher level of COVID-19-related anxiety and increase in depression. These findings are interesting considering the fact that in comparison to earlier studies on HS showing that HS were less educated, and/or less healthy physically and/or mentally (e.g., [20, 27]), our participants were young during the war (mainly children or adolescents), and therefore, many of them were less likely to be aware of and/or remember all of the traumatic events they lived through, which may lead to the assumption that they would be less susceptible to effects of new threatening life events, however this is not what we identified. Our results also lend support to other findings regarding Corona-related negative mental health effects among HS in Israel, especially among those who suffered during or after WWII from infectious diseases or who report experiencing PTSD symptoms [32, 33], possibly because the threat of the disease to life and the imposed social restrictions trigger early memories difficult to bear. In general, our findings indicate that exposure of HS to prolonged negative life events such as the COVID-19 pandemic is more likely to lead to more adverse outcomes in mental health than in mentally stronger social groups.

Despite this vulnerability among the HS, both study groups were similar in their WTL, which is a unique and valuable indicator of general well-being, due to the commitment to fight for life embedded within WTL with its instinctive and psychological components [31, 34]. This finding is especially important considering that WTL has also been shown as a predictor of long-term survival among older adults while controlling for various relevant characteristics such as age and health status [35, 36]. In other words, despite their relative vulnerability due to past trauma, a lower level of education and self-perceived health, the HS in our study demonstrated similar resilience to that of other older adults while coping with the unexpected threatening and long-lasting event of the COVID-19 pandemic. Although assessed differently, the coexistence of both vulnerability and resilience has been reported in previous studies conducted in Israel (e.g., [18, 27]), probably because the HS that are still alive have developed specific psycho-behavioral coping patterns such as daily planning and activity engagement which was reported to reduce psychological distress during the Coronavirus pandemic even among HS with high PTSD levels [37], and may explain to some degree the ability of HS to reach old age in spite of their traumatic pasts.

Regarding the entire sample, over 70% of the participants reported experiencing COVID-19-related anxiety (to a very great/great/some extent), and close to 20% reported an increase in depression (27% among the HS and 15% among the other group). Increase in depression is especially problematic in old age due to its association with other chronic diseases and mortality (e.g., [38]). In addition, 22% of our older participants responded that their WTL weakened during the pandemic (25% of the HS and 20.2% of the other group), indicating that among some, the desire to continue living may weaken in the face of difficult physical, economic, or social life conditions, similarly to previous reports [31, 34].

Considering that data collection for this study began rather early in the development of the pandemic (most of the interviews were conducted immediately after the first enforced closure) and was completed approximately five months before the end of the most severe period of the first wave of the pandemic in Israel, it could be assumed that the percentage of people reporting such negative outcomes has further increased over time among older adults in Israel. This assumption, along with a previous finding among older Israelis indicating that WTL precedes the occurrence of depression over time [39], and the well-established causal association between depression, physical health and mortality (e.g., [38]) lead to the conclusion that the increasing pandemic-related needs of older population groups should be addressed before there is further deterioration in their health and well-being.

Our multivariate analyses indicate that being a HS is not a statistically significant explanatory variable of COVID-19 anxiety, probably because of the small size of our sample and the significantly lower number of our participants in the HS’ group in comparison to the non-HS group. Additionally and logically, other indicators of mental health such as increase in depression, are more powerful in explaining the emotional reaction of COVID-19 anxiety. Among the other older Israelis, in addition to an increase in depression (Beta = 0.231), decline in WTL (Beta = 0.149) and, to a lesser degree, being a woman (Beta = 0.138) also contributed to the explanation of the COVID-19 anxiety. Regarding gender differences, our finding is similar to those of numerous previous reports showing that women suffer from depression more than men (e.g., [38, 40]). In sum, the results regarding COVID-19 anxiety, increase in depression and decline in the WTL indicate that the current pandemic, which can be considered a prolonged stressful life event, has negatively affected mental health and subjective well-being among older adults living in Israel including HS.

### Limitations of the study

The cross-sectional design of the current study prevents us from reaching causal conclusions between COVID-19 anxiety and the increase in depression or decline in WTL. In addition, budget constraint and the COVID-19 restriction of abstaining from social interaction prevented us from investigating a larger sample, a wider range of items for evaluating each variable, and from conducting face-to-face interviews, all of which would have been more reliable than our brief telephone interviews. Data based on the questions regarding depression do not enable us to assess new cases of depression occurring during the Corona virus pandemic. We assumed that it was generally too soon to develop depression as a result of the pandemic at the time of initial data collection (beginning of June, 2020, which was a rather early stage of the pandemic (April 12^th^, was the start of the first full shutdown enforced by the Israeli government which was gradually opened during later weeks)), and most data were collected during the first month of the study. Additionally, although we recruited participants from all over the nation, this study is not based on a representative sample of the Israeli older adult population. However, our results are in line with those of previous comparative studies in Israel between HS and control groups regarding concurrent vulnerability and resilience among the HS (e.g., [18, 27]), and with Israeli studies about the psychological negative effects of the Corona pandemic especially on HS [32, 33]. The results also generally support the life-events, stress, coping and health theory by showing the decline in mental health and well-being during the first months of the pandemic. The consistency between our findings and those of previous studies contributes to the validity of this study.

## Conclusions

Our findings support the life events, stress, coping and health theory, by showing that HS seem to be more mentally vulnerable to the COVID-19 pandemic and probably to any national crisis, than other older adults living in the same country. Nevertheless, HS concomitantly show resilience in their general well-being and in their inner drive to fight for life, similar to that of other older people. Regarding the explanation of the occurrence of COVID-19 anxiety, the HS’ status is not a significant factor while other emotional conditions such as increase in depression and decline in WTL are the factors that best explain occurrence of COVID-19 anxiety during the first months of the pandemic. Regarding the entire sample, a relatively high percentage of the participants (above 70%) reported experiencing COVID-19 anxiety, and about 20% reported an increase in depression or decline in the WTL as a result of the COVID-19 pandemic. These results lead us to suggest that with the prolongation of the pandemic, the reported decline in mental health and well-being may cause further deterioration in health among the aged in general and particularly among socially more vulnerable population groups. The current results, the associations between depression and weakening in WTL with morbidity and even mortality reported in the literature, and the finding that decline in WTL precedes the occurrence of depression, all lead us to conclude that maintaining WTL and strengthening WTL can protect people from further decline in their mental health. All of these, and the potential negative developments due to the prolongation of this crisis, must raise societal attentiveness and concern. Policy makers, service providers and practitioners should pay special attention to older people in general, and particularly to the more vulnerable population groups among them such as HS, especially during difficult times. This may require developing additional professional practices and outreaching interventions directed to appropriately locating and addressing mental, physical and social needs. For instance, interventions to strengthen daily planning and activity engagement have been reported to reduce psychological distress during the Corona pandemic even among HS with high PTSD levels [37]. Additionally, intervention programs should include practices for maintaining and strengthening existing psychosocial protective resources such as the will to live.

## Data Availability

The datasets generated and/or analysed during the current study are not publicly available due to the authors currently being in the process of analysing the data for future publications, however the datasets are available from the corresponding author on reasonable request.

## Abbreviations

HS: Holocaust survivors
Non-HS: Non-survivors. Participants who were not Holocaust survivors
WTL: Will to live
